# Occipitosacral Fusion for Multiple Vertebral Fractures with Kyphotic Deformity in a Patient with Mutilating Rheumatoid Arthritis: A Case Report

**DOI:** 10.1055/s-0037-1601321

**Published:** 2017-03-20

**Authors:** Tetsu Tanouchi, Takachika Shimizu, Masatake Ino, Naofumi Toda, Nodoka Manabe, Kanako Itoh, Keisuke Fueki

**Affiliations:** 1 Department of Orthopedic Surgery, Gunma Spine Center, Harunaso Hospital, Takasaki, Gunma, Japan

**Keywords:** Kyphosis, osteoporosis, rheumatoid arthritis, thoracolumbar spine, vertebral compression fracture

## Abstract

Osteoporotic vertebral fractures are well-known complications of rheumatoid arthritis. The management of multiple vertebral fractures with kyphotic deformity is controversial. We present a case of a patient with mutilating rheumatoid arthritis who had multiple vertebral fractures with kyphotic deformity after occipitothoracic fusion for rheumatoid cervical disorder. Occipitosacral fusion was effective to create stable spine with better sagittal alignment in this case, but careful clinical assessment for early detection and management of postoperative insufficient pelvic fracture were required.

Osteoporosis and osteoporotic vertebral compression fractures are well-known complications of rheumatoid arthritis (RA). 1 2 3 4 Especially, patients with mutilating-type RA who often receive long-term glucocorticoids treatment and undergo multiple surgeries are in a high-risk group of vertebral fractures. 5 6 In addition, multiple vertebral fractures can be associated with functional disability, 7 8 and increased morbidity and mortality due to severe pain and deformity. 9 10 Therefore, it is critical to manage pain and prevent the progress of the deformity to reduce serious complications through treatment of osteoporotic vertebral fractures.

We report a case of a patient with mutilating-type RA who treated by occipitosacral fusion for multiple vertebral fractures with kyphotic deformity.

## Case Report

**Fig. 1:**
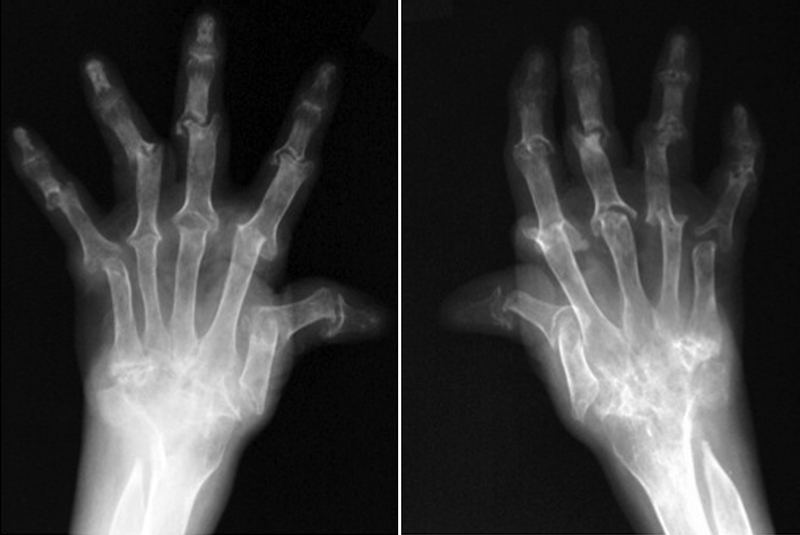
X-ray of both hands showed typical mutilating changes.

**Fig. 2:**
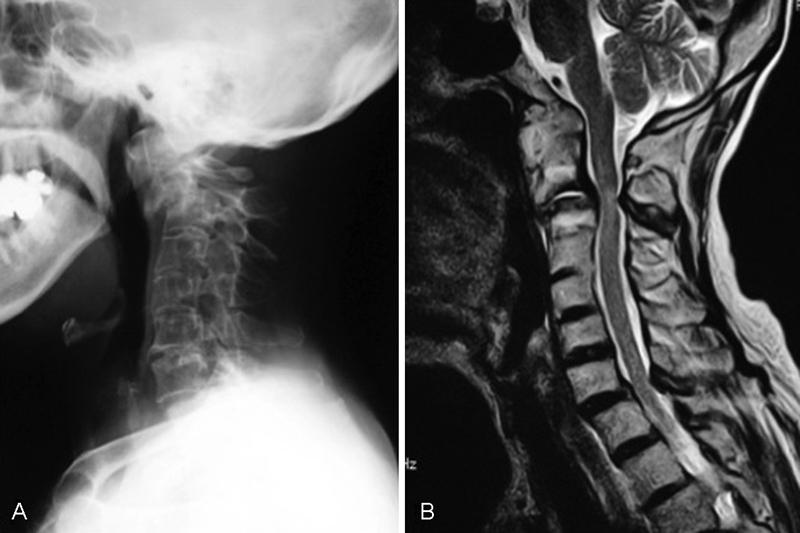
( **A** ) Lateral radiography of the cervical spine revealed ankylosis at craniovertebral junction and anterior subluxation at C2–C3. ( **B** ) Magnetic resonance imaging demonstrated marked severe compression of the spinal cord at C2–C3 and moderate compression at the cervicothoracic junction.

*Patient history* : a 58-year-old woman had RA with typical mutilating-type joint involvements for 30 years and was treated with oral glucocorticoid (5 mg) and methotrexate (6 mg) ( Fig. 1 ). In 2003, she rapidly presented with gait disturbance and upper extremity functional disturbance and visited our hospital. Her neurologic status, as determined by the Ranawat classification system, was class IIIB. Lateral radiography of the cervical spine revealed ankylosis at the craniovertebral junction and anterior subluxation at C2–C3. Magnetic resonance imaging (MRI) demonstrated marked severe compression of the spinal cord at C2–C3 and moderate compression at the cervicothoracic junction. Lateral radiography of the upper thoracic spine revealed stepladder deformity; however, there was no vertebral column fracture in the thoracic and lumber spine ( Fig. 2 ).

**Fig. 3:**
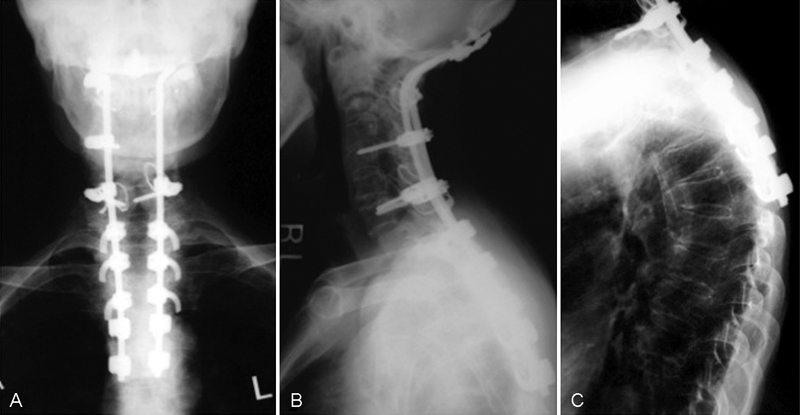
( **A,B** ) Anteroposterior and lateral radiography of the cervical spine after O–T5 fixation. After the first surgery, anterior displacement of C2 on C3 was completely reduced. ( **C** ) However, vertebral fracture of T5 at the lowest level of the fusion area developed.

*Operation 1* : the patient underwent occipitothoracic fusion (O–T5 fusion) using RRS Loop Spinal System (Robert Reid Inc., Tokyo, Japan) with multiple thoracic hooks ( Fig. 3A, B ). Autologous iliac crest grafts were used as fusion substrate. Successful cervical realignment with complete reduction of C2 was obtained. One month after surgery, she had a vertebral fracture of T5 at the lowest level of the fusion area ( Fig. 3C ). This adjacent level fracture had not worsened and was cured with no complaint by conservative treatment. Preoperative neurologic deficits had improved and she started to walk (Ranawat IIIA). She maintained daily activity for 2 years following this surgery.

**Fig. 4:**
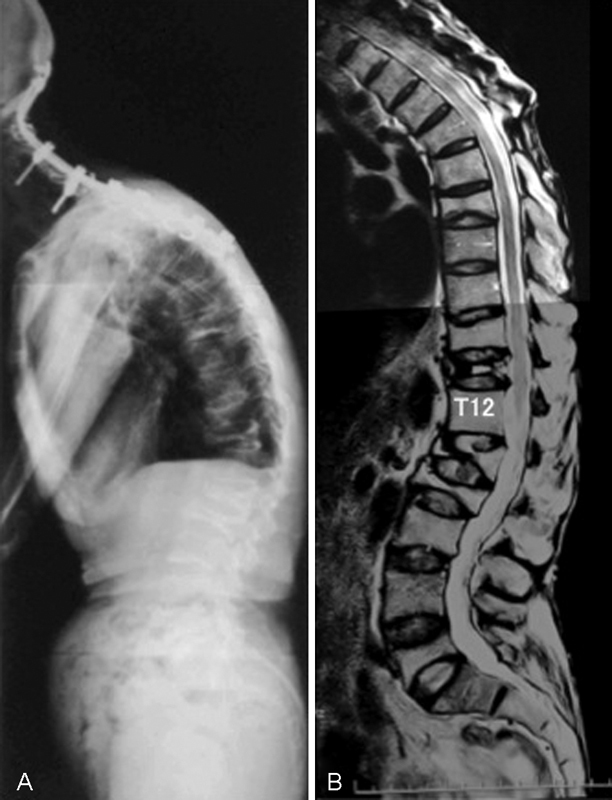
( **A** ) Lateral radiography of the whole spine and ( **B** ) T2-weighted magnetic resonance imaging revealed multiple vertebral fractures (T5, 7, 10, 11, L1, 2, 3, 5) and osteonecrosis of T11.

*Multiple vertebral fractures* : 2 years after first surgery, she had a vertebral fracture of L1 without trauma. With this fracture as a start, she had multiple vertebral fractures of T7, 10, 11, L1, 2, 3, and 5 during 4 months, and thoracolumbar kyphotic deformity has progressed rapidly ( Fig. 4A, B ). In addition, the fracture of T11 landed to vertebral osteonecrosis. Her clinical symptoms were severe back pain exacerbated by movement, difficulty in looking straight, and appetite loss due to a symptom of gastroesophageal reflux disease (GERD). This symptom of GERD was unique in that she could eat and swallow easily only when she sat on a reclining easy chair to decrease her kyphosis.

**Fig. 5:**
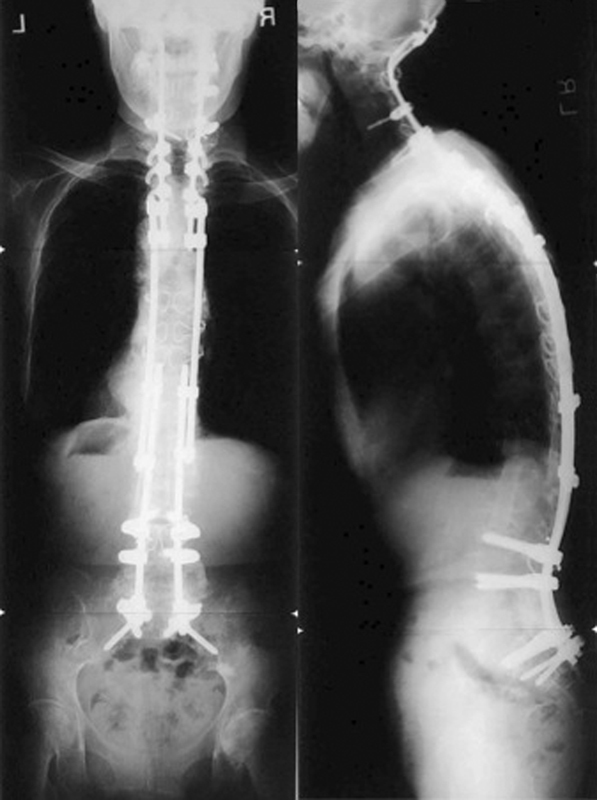
Anteroposterior and lateral radiography of the whole spine after occipitosacral fusion. Anterior fibula strut bone graft was also done at T10–T12 and T12–L3.

*Operation 2* : In 2006, she underwent posterior and anterior combined correction surgery for her sagittal spinal deformity ( Fig. 5 ). At first, posterior-instrumented correction and fixation from T5 to sacrum was performed, connecting to occipitothoracic instrumentation. S1 pedicle screws and S2 alar screws were used as distal anchors. Autologous iliac crest grafts were used as fusion substrate. Secondary, 4 weeks later, fibula strut bones were grafted from the anterior at the T10–T12 and T12–L3 levels. A hip spica hard corset was worn for 3 months to support the correction of kyphotic deformity. Her standing and walking with one crutch became stable again because of decreased back pain and improved sagittal alignment after surgery. Her gastrointestinal obstruction also improved. She achieved solid fusion at 1 year after surgery.

**Fig. 6:**
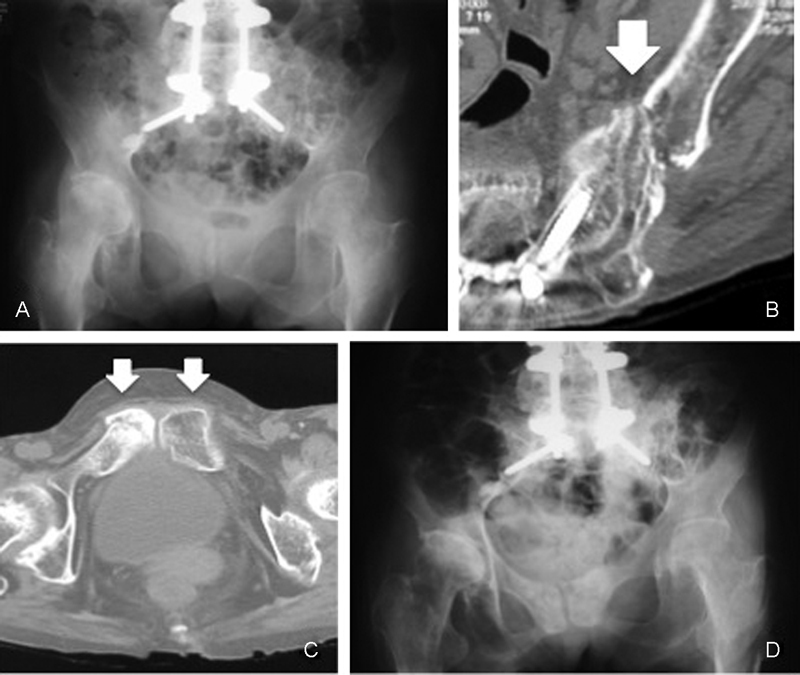
( **A** ) Pelvic X-ray showed no abnormal findings. ( **B** ) Axial computed tomography (CT) images of the left sacrum and ilium showed linear fracture gaps of reaching to iliac bone harvesting sight. ( **C** ) Axial CT images of the bilateral pubis showed fracture lines. ( **D** ) Posttreatment pelvic X-ray showed oval deformity of the pelvic ring.

*Pelvic insufficiency fracture* : 15 months after final surgery, she suddenly suffered from severe buttock pain without a history of trauma, and she could not even sit and turn over herself, but pelvic X-ray showed no abnormal findings. Pelvic computed tomography (CT) revealed fractures in left sacral ala parallel to the sacroiliac joints through to posterior superior iliac spine from where bone was harvested. T2-weighted MRI demonstrated high signal region around left sacroiliac joint. Judging from the results of these examinations, the patient was diagnosed as having insufficiency fracture of the sacral and sacroiliac joint ( Fig. 6A–C ). She recovered with bed rest followed by light physical therapy for 6 weeks and achieved full weight-bearing 3 months after the injury ( Fig. 6D ). She gradually returned to her activity. Eight years after this pelvic complication, she died of pneumonia at the age of 70 years, and just before her death, she maintained her daily activity without spine and pelvic trouble.

## Discussion

This patient was a mutilating-type RA as most aggressive type RA, and she had severe destructive cervical disorder with progressive myelopathy. For such severe destructive cervical disorders, we have performed O–T fusion and reported clinical results and complications. 11 12 In our previous report, distal vertebral fractures were most frequent complication, and most of distal vertebral fractures were cured by conservative treatment like this patient.

Two years later after the episode of adjacent level vertebral fracture, seven vertebral fractures of thoracic and lumber spine occurred without a history of trauma in less than half a year. These fractures were thought to be different from adjacent trouble after O–T fusion and to be correlated with a vicious cycle of osteoporotic vertebral fracture and kyphosis. It was difficult to control this vicious cycle.

Osteoporotic vertebral compression fractures are well-known complications of RA, and the risk of spine fracture in patients with RA has been reported to be more than six times higher than that in the non-RA population. 1 Some researchers reported the clinical results of treatments for individual vertebral fractures. 13 14 15 However, few studies have described the treatment of the multiple vertebral fractures with severe kyphotic deformity of rheumatoid patients. They might not get medical attention as a result of their low-activity life. In this report, she had severe back pain due to osteonecrosis of T11, appetite loss due to symptom of GERD derived from kyphotic deformity, and her strong motivation for surgical treatment. Thus, she decided to undergo aggressive surgery.

When surgeons perform long fixation surgery for severe osteoporotic patients, it is important to use a combination of several types of anchors such as pedicle screw, sublaminar wiring, and hooks. Moreover, they have to do careful postoperative management such as orthosis and physical therapy.

In our patient, however, delayed pelvic insufficiency fracture resulted in worsening of daily activity. This fracture was difficult to diagnose on even CT and MRI because of osteoporosis and beam-hardening artifact. 16 17 18 Thus, it is important to provide careful clinical assessment for early detection and management of this complication.

When we performed C0–S fusion, we selected a combination of S1 pedicle screws and S2 alar screws as distal anchor to avoid iliac screw trouble due to destruction of hip joints in future. Now we consider to avoid bone harvesting from posterosuperior iliac spine and to use iliac screw prophylactically in these patients.

We have demonstrated the technical feasibility of performing occipitosacral fusion in rheumatoid patient with kyphotic deformity due to multiple vertebral fractures. This treatment is definitely invasive and challenging surgery, but it has great potential for improving several symptoms due to vertebral fractures and kyphotic deformity.
